# *In situ *studies of algal biomass in relation to physicochemical characteristics of the Salt Plains National Wildlife Refuge, Oklahoma, USA

**DOI:** 10.1186/1746-1448-1-11

**Published:** 2005-12-15

**Authors:** Kelly M Major, Andrea E Kirkwood, Clinton S Major, John W McCreadie, William J Henley

**Affiliations:** 1 Department of Biological Sciences, University of South Alabama, Mobile, AL 36688 USA; 2 Department of Botany, Oklahoma State University, Stillwater, OK 75078 USA

## Abstract

This is the first in a series of experiments designed to characterize the Salt Plains National Wildlife Refuge (SPNWR) ecosystem in northwestern Oklahoma and to catalogue its microbial inhabitants. The SPNWR is the remnant of an ancient ocean, encompassing ~65 km^2 ^of variably hypersaline flat land, fed by tributaries of the Arkansas River. Relative algal biomass (i.e., chlorophyll concentrations attributed to Chlorophyll-*a*-containing oxygenic phototrophs) and physical and chemical parameters were monitored at three permanent stations for a one-year period (July 2000 to July 2001) using a nested block design. Salient features of the flats include annual air temperatures that ranged from -10 to 40°C, and similar to other arid/semi-arid environments, 15–20-degree daily swings were common. Shade is absent from the flats system; intense irradiance and high temperatures (air and sediment surface) resulted in low water availability across the SPNWR, with levels of only *ca*. 15 % at the sediment surface. Moreover, moderate daily winds were constant (*ca*. 8–12 km h^-1^), sometimes achieving maximum speeds of up to 137 km h^-1^. Typical of freshwater systems, orthophosphate (PO_4_^3-^) concentrations were low, ranging from 0.04 to <1 μM; dissolved inorganic nitrogen levels were high, but spatially variable, ranging from *ca*. 250–600 μM (NO_3_^- ^+ NO_2_^-^) and 4–166 μM (NH_4_^+^). Phototroph abundance was likely tied to nutrient availability, with high-nutrient sites exhibiting high Chl-*a *levels (*ca*. 1.46 mg m^-2^). Despite these harsh conditions, the phototrophic microbial community was unexpectedly diverse. Preliminary attempts to isolate and identify oxygenic phototrophs from SPNWR water and soil samples yielded 47 species from 20 taxa and 3 divisions. Our data indicate that highly variable, extreme environments might support phototrophic microbial communities characterized by higher species diversity than previously assumed.

## Background

Relatively few microbial communities in hypersaline ecosystems, i.e., those with salinities consistently greater than that of seawater (35 psu), have been described in detail. Most investigations have focused on coastal salterns [24] and sabkhas [6,7], or inland, chronically hypersaline lakes such as the Dead Sea [17], Great Salt Lake [22] and Mono Lake [13]. Moreover, such studies have targeted planktonic and/or submerged benthic microbial mat communities that tend to be persistent and are millimeters to centimeters in thickness.

In contrast, the Salt Plains National Wildlife Refuge (SPNWR) in northwest Oklahoma, U.S.A. features extensive subaerial flats that are permanently moistened by hypersaline groundwater seepage. We would characterize this habitat as athalassic [12] because, although the salt source exists as buried Permian marine deposits, there is no geologically recent connection to any marine system. The nearest contemporary marine system is the Gulf of Mexico at a distance of 900 km. The SPNWR flats generally lack visible photoautotroph biomass, yet we have been able to detect chlorophyll and isolate viable oxygenic phototrophs from nearly every soil and water sample collected.

Herbst [12] divides saline systems into four categories based on axes of habitat stability and salinity stress: stable lakes of low salinity, stable lakes of moderate to high salinity, temporary lakes of low to moderate salinity and extreme ephemeral hypersaline waters. The last of these appears to approximate the condition of the SPNWR flats, however, the reported dominance of microbial halophiles in such habitats does not adequately describe the broad range of salinity tolerances among the many taxa we have isolated to date [4, Kirkwood & Henley, submitted]. Thus, the SPNWR, a semi-terrestrial ecotone, appears to be different from any hypersaline microbial habitat studied to date. In this paper, we report on the physicochemical conditions of the SPNWR ecosystem and relate them to temporal and spatial variation in chlorophyll concentrations (i.e., approximate estimates of biomass attributed to all Chlorophyll-*a *containing oxygenic phototrophs) across the flats.

## Results

### Climatological and soil temperature data

The SPNWR can be characterized as a formidable habitat, particularly during the summer months. Climate data for the 2000–2001 study period indicate that phototrophic microbial inhabitants of the flats are subject to broad daily and seasonal swings in temperature, light and wind speed (Table 1). Average incident solar radiation levels were lowest in winter and highest in summer, ranging from *ca*. 9 to 28 MJ m^-2^. This difference was attributable to a combination of daylength and maximum midday solar flux, that were higher (*ca*. 53% and 76%, respectively) on the June than the December solstice (data not shown). Average day/night temperatures exhibited the largest degree of variation in summer and early fall (July–September), resulting in *ca*. 15-degree differences between daytime and nighttime hours. Average daily air temperatures hovered around 30°C from July through September. In general, rainfall totals were lowest in summer and winter months, with maxima occurring in October 2000 (18.03 cm) and March, May and June of 2001 (>6 cm). Average daily winds were of moderate speeds and ranged from *ca*. 12 to 19 km h^-1 ^across the flats, with daily maxima ranging from 54 to 137 km h^-1 ^(Table 1).

**Table 1:** Climatological data from July 2000 through July 2001 for Cherokee, Oklahoma.

Month	Temperature (°C)			Rainfall (cm)	Wind Speed (km h^-1^)		Solar Radiation (MJ m^-2^)
2000–2001	min	max	mean	monthly total	max	mean	mean
Jul	21.1	35.0	27.9	5.33	137.4	16.3	27.07
Aug	22.8	39.4	31.1	0.51	54.1	16.6	25.05
Sep	15.6	33.3	24.7	0	60.3	17.5	21.16
Oct	11.7	22.8	17.0	18.03	73.5	15.3	12.96
Nov	0	11.1	4.8	3.30	63.4	12.7	9.99
Dec	-7.2	2.8	-2.3	1.78	79.5	13.4	8.99
Jan	-3.3	6.7	1.2	1.78	78.8	13.2	9.87
Feb	-2.8	8.3	2.4	8.38	81.7	14.5	9.92
Mar	1.7	12.8	6.9	6.10	85.8	12.1	15.26
Apr	8.9	23.3	16.7	1.02	100.2	18.5	20.89
May	13.9	27.2	20.6	6.10	100.6	14.6	24.20
Jun	18.9	33.3	26.1	6.60	98.1	18.5	27.99
Jul	24.4	39.4	32.0	0.25	67.4	18.0	27.49

(MESONET: The Oklahoma Climatological Survey)

The modeled T_soil _for June through September 2001 is shown in figure 2. The median and maximum diel amplitudes of fluctuation in T_soil _were 22.4 and 30.6°C; only overcast days exhibited amplitudes <15°C. Frequency distributions (5°C intervals) of modeled T_soil _(all values and daily maxima) are shown in figure 3. The median predicted T_soil _for these four months was 30.6°C, and 22% of the values exceeded 40°C. Daily maxima T_soil _were skewed, with approximately 80%, 51% and 20% of the days reaching at least 40, 45 and 50°C, respectively (Fig. 3). In July and August only, predicted maximum T_soil _exceeded 45°C on 53 days (85%) and 50°C on 24 days (39%). The highest modeled T_soil _was 53°C, whereas, the observed maximum was 55.5°C on two days in early August. Taken together, the high degree of both daily and seasonal variability in air/soil temperature, light and wind speed represent what might be defined as the most *extreme *of the extreme environments (i.e., an environment characterized by high variability and multiple stressors).

**Figure 1:**
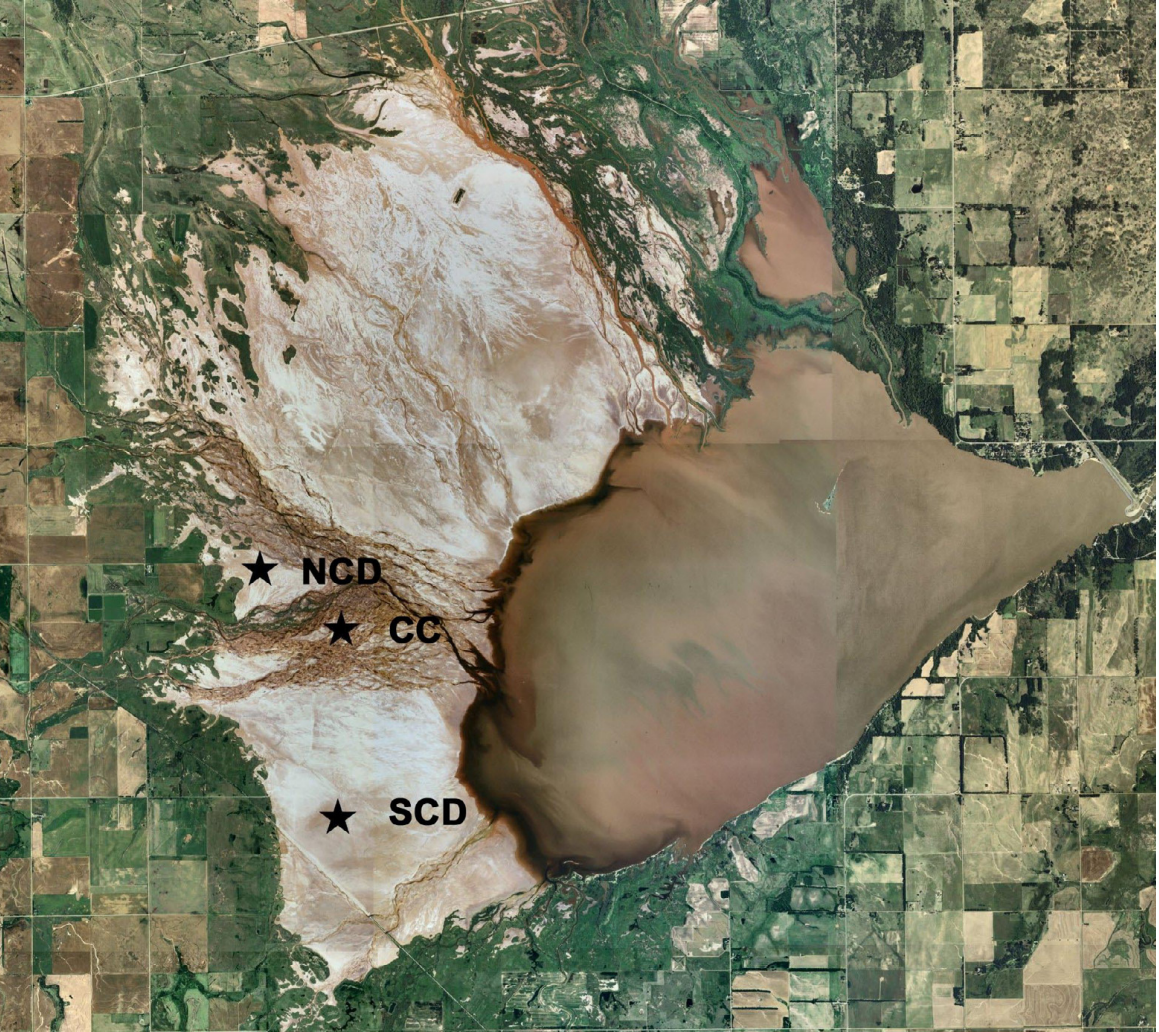
Aerial view of the Salt Plains National Wildlife Refuge (SPNWR) depicting tributaries of the Arkansas River, the reservoir and salt flats. Labels denote North Crystal Dig (NCD), Clay Creek (CC) and South Crystal Dig (SCD) sampling stations. (This image was extracted from the 2003 NAIP aerial image database (Alfalfa Co., OK); http://www2.ocgi.okstate.edu/2003img1/)

**Figure 2:**
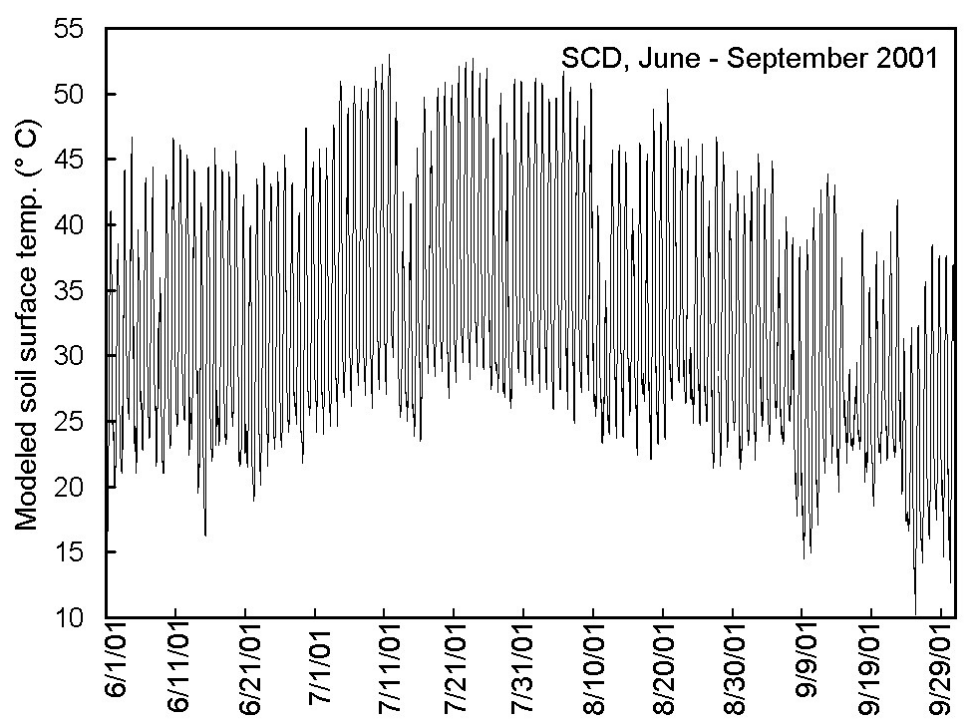
Modeled T_soil _at SCD for June through September 2001.

**Figure 3:**
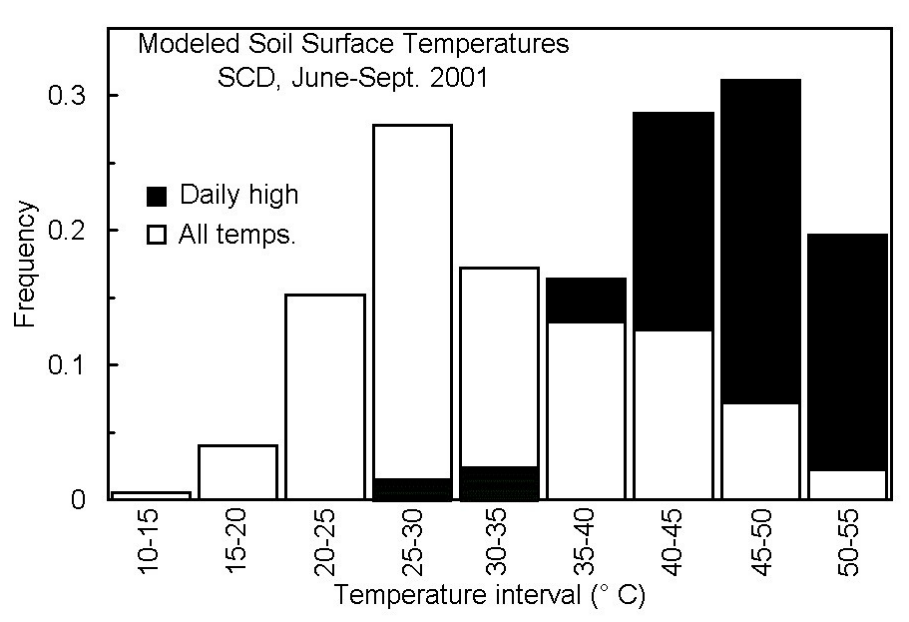
Frequency distributions (5°C intervals) of modeled SCD T_soil _(open bars) and daily maxima (filled bars) derived from figure 2.

### Algal diversity and biomass

The SPNWR supports a surprisingly diverse phototrophic microbial community. To date, 47 species of oxygenic phototrophs from 20 taxa and 3 divisions (*Chlorophyta*, *Cyanophyta *and *Bacillariophyta*) have been isolated and identified from SPNWR soil and water samples (Table 2). The majority of species identified (62%) belong to the prokaryotic division of cyanophytes. Not unexpectedly, four green algal species belong to the halophilic genus, *Dunaliella*. However, unlike the salt-adapted organisms typical of high salinity environments, many of the salt plains isolates exhibit a wide range of salt tolerance and some are able to grow in freshwater to at least 150 psu (Kirkwood & Henley, submitted).

**Table 2:** Summary of algal taxa isolated from the Salt Plains National Wildlife Refuge, Oklahoma.

Taxonomic Division	Genus	No. of species	No. of isolates	Sample Types	Isolation Salinities (psu)	Isolate Distribution
Chlorophyta	*Dunaliella*	4	27	soil, brine pools	50, 100	south, central, north,
Chlorophyta	*Tetracystis*	1	1	dried algal mat	10	central
Chlorophyta	*Tetraselmis*	1	1	soil	50	central

Cyanophyta	*Aphanocapsa*	1	1	dried algal mat	10	central
Cyanophyta	*Aphanothece*	2	2	dried algal mat	10	central
Cyanophyta	*Cyanodictyon*	1	2	soil, brine pools	50	central, north
Cyanophyta	*Geitlerinema*	7	31	soil	10, 50, 100	central
Cyanophyta	*Halomicronema*	1	1	soil, algal mat	50	central
Cyanophyta	*Komvophoron*	1	2	soil	10	central, south
Cyanophyta	*Leptolyngbya*	1	1	soil	50	north
Cyanophyta	*Lyngbya*	1	2	soil	50	central
Cyanophyta	*Phormidium*	8	27	soil, algal mat	10, 50, 100	central
Cyanophyta	*Pseudanabaena*	3	10	soil	10, 50	central
Cyanophyta	*Spirulina*	1	2	pool sediment	10, 50	central
Cyanophyta	*Tychonema*	2	3	algal mat	10	central

Bacillariophyta	*Amphora*	4	38	soil, brine pools	10, 50, 100	south, central, north
Bacillariophyta	*Bacillaria*	1	1	soil	50	central
Bacillariophyta	*Eucocconeis*	2	4	soil	10, 50	south, central, north
Bacillariophyta	*Navicula*	5	18	soil	10, 50, 100	south, central, north
Bacillariophyta	*Nitzschia*	1	3	soil	10, 50	central

**Figure 4:**
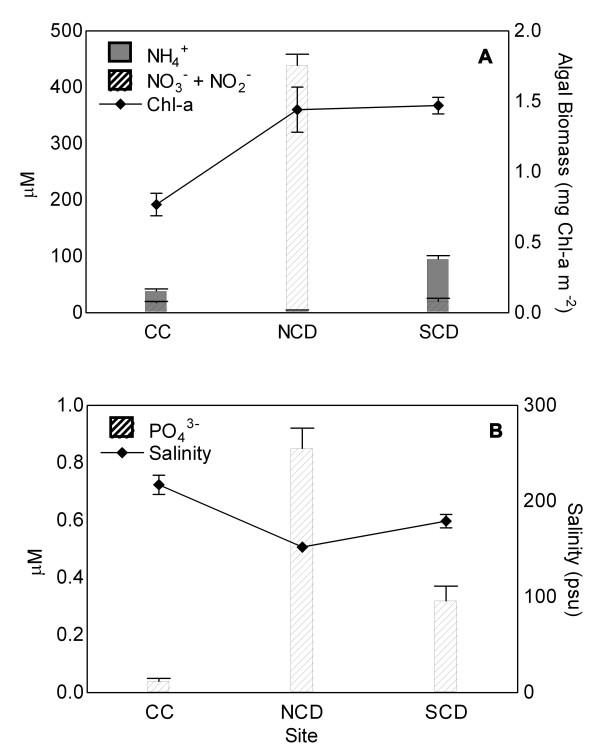
Variation in algal biomass as a function of (A) nitrogen, NH_4_^+ ^and NO_3_^- ^+ NO_2_^-^, (B) orthophosphate, PO_4_^3- ^and ground water salt concentration per site. Error bars denote SE of means. (Chl-*a*: n = 108; NH_4_^+^, NO_3_^- ^+ NO_2_^-^, PO_4_^3- ^and salinity: n = 34–36)

Although no clear seasonal trends in algal biomass (i.e., biomass attributed to Chl-*a*-containing oxygenic phototrophs), nutrient concentration(s) or salinity were noted over the one-year study period, site-specific differences were evident. In terms of algal biomass, Chl-*a *concentration was significantly lower at the CC site (0.77 mg m^-2^) relative to either the NCD or SCD sites (*ca*. 1.46 mg m^-2^; P = < 0.001; Fig. 4A). Nitrogen and phosphorus concentrations were higher at both the NCD and SCD sites when compared to those at the CC site (Fig. 4A,B). However, the dominant form of nitrogen varied with site such that NCD exhibited unusually high amounts of NO_3_^- ^+ NO_2_^-^, ranging in concentration from *ca*. 250 to nearly 600 μM (i.e., on average, almost 23 times more than NO_3_^- ^+ NO_2_^- ^concentrations at either the SCD or CC sites), while the SCD site exhibited high concentrations of NH_4_^+^up to 166 μM (Fig. 4A). The average NH_4_^+ ^concentrations were significantly lower at the CC and NCD sites (38.4 and 4.3 μM, respectively) relative to those at the SCD site (P = < 0.001). On the whole, soluble reactive phosphorus (SRP) availability was very low across the flats, ranging from 0.04 to 1.7 μM; PO_4_^3- ^concentrations were often below the level of detection (i.e. < 0.01 μM). Levels of SRP were significantly lower at the CC site (0.04 μM), with moderate and high levels noted for the SCD and NCD sites (0.32 and 0.85 μM, respectively; P = < 0.001; Fig. 4B). Thus, algal abundance (i.e., biomass of oxygenic phototrophs) appears to be driven by the availability of nitrogen (either in the form of NH_4_^+ ^or NO_3_^- ^+ NO_2_^-^) in combination with relatively low concentrations of orthophosphate in the SPNWR system (Fig. 4A, B); statistical analyses did not support a clear correlation between N and P at the level of P < 0.05. Average ground water salinities ranged from 152 psu at SCD to 218 psu at CC, with that of NCD being mid-range at 178 psu (Fig. 4B). Although ground water salinity was significantly different among all sites (P = < 0.001), soil moisture content at the sediment surface (*ca*. 15%) did not vary among sites. Thus, it seems that nutrient availability is biologically more important than salt and/or freshwater availability in this system. Despite the fact that Discriminant Function Analysis aligned CC and SCD together according to physical and chemical similarities (i.e., NCD was the outlier in this study; data not shown), algal biomass was high at stations with high N and P availability (i.e., NCD and SCD).

## Discussion

To our knowledge, with the exception of some early qualitative descriptions of the plant communities of the region (e.g., Ortenburger and Bird [18]; Penfound [20] and Williams [30]), the first detailed description of the vegetation of the Salt Plains National Wildlife Refuge was provided by Baalman in 1965 [3]. Baalman documented the vegetational history and provided a higher plant species list for lands adjacent to the salt flats, with occasional anecdotal references to observations of cyanophytes. Similar to Baalman's work, Unger [27,28] provided further documentation of the distribution of salt-tolerant higher plant species for the region as a function of soil characteristics, specifically, salinity. Since the completion of Unger's work in 1968, little else has been done to describe the salt flat ecosystem and, in particular, the unique phototrophic microbial communities that inhabit it. The present investigation provides the first detailed account of the physicochemical profile of the Oklahoma salt flat system and a preliminary sampling of photosynthetic microbes that inhabit the SPNWR.

Generally, high salinity is the norm in this ecosystem; low salinities coincide with rainfall as freshwater runs over the flats. In summer, this semi-arid ecosystem is hot and dry with salt crystals covering the sediment surface. It is not unusual for air temperatures to reach 40°C. However, surface temperatures of the flats can be considerably higher or lower, depending upon solar heating and/or evaporative cooling. Furthermore, shade cover is absent from this system; organisms living on the flats are routinely exposed to full-sunlight and high levels of UV radiation. Thus, the SPNWR is an extreme environment with phototrophic microbial inhabitants that experience broad changes in temperature, salinity and irradiance that occur over daily, as well as, seasonal time scales. Moreover, the flats are heterogeneous, exhibiting spatial variability in salinity and nutrient availability, and hence, algal biomass. Such habitats, exhibiting major shifts between low and high extremes at regular, irregular and/or episodic intervals, are typically thought to be low in species diversity [8]. Contrary to previous studies addressing the effect of salinity on microbial community structure (e.g., Herbst and Blinn [11]), our data and those of Henley and Kirkwood (submitted) indicate that extreme habitats have the potential to support diverse phototrophic microbial communities. Similarly, Williams [31] also concluded that salinity is less important in determining biological community structure than once thought.

Many of the 47 species of oxygenic phototrophs listed herein are tolerant of broad ranges in salinity and temperature [10, Kirkwood and Henley, submitted, Major and Henley, in prep.]. Unlike true salt-adapted halophiles that thrive in high-salt environments, we suggest that the resident oxygenic phototrophs of the SPNWR fit the definition of poikilotrophic microbes (*sensu *Gorbushina and Krumbein [8]). This is to say, that these phototrophs are adapted to extreme, often rapid, environmental change. On the whole, phototrophic microbes of the SPNWR tend to be present in low numbers and exhibit relatively slow growth rates [10, Major and Henley, in prep.], typical of microbes living in high-stress environments [8]. Nutrient availability (i.e., inorganic N, regardless of form, and PO_4_^3-^) plays an important role in determining phototrophic microbial biomass distribution across the flats, with high nutrient concentrations having a positive influence on abundance. Because orthophosphate levels are very low, P is likely present in other, less biologically available forms. Low Chl-*a *concentrations associated with the Clay Creek site might be due to frequent freshwater inflow and, consequently, low nutrient availability and chronic scouring as this site resides in the center of the tributary system that flows across the reserve (Fig. 1).

As Williams [32] points out, saline habitats are important natural assets of considerable economic, ecologic, scientific and conservation value. Most notably, their unique physical and chemical characterization and distinctive biota set them apart from other aquatic and/or semi-aquatic ecosystems. Extreme environments and the microbes that inhabit them offer invaluable insight into the fundamental physiological and ecological mechanisms of stress tolerance. Furthermore, such avenues of research might contribute to our understanding of early evolution and knowledge of agricultural practices in saline soils. Hypersaline microbial communities represent a largely untapped resource for potentially unique economically and scientifically useful model organisms.

## Methods

### Study site

The Salt Plains National Wildlife Refuge (SPNWR) resides in Alfalfa County in northwestern Oklahoma where salt flats cover approximately 64 km^2 ^([14]; Fig. 1). Farm and cattle ranching lands border the flats on three sides, while Great Salt Plains Reservoir is located at the southeastern edge of this system. Tributaries of the Arkansas River intermittently flow over the flats to feed the reservoir. Three permanent high-salinity sampling stations were established on the flats in July of 2000, each characterized by ground water salinities and soil moisture content of *ca*. 125–250 psu and 15%, respectively. Because this project was designed to specifically target extremophilic algae (i.e., oxygenic phototrophs), site selection was deliberately relegated to high salinity areas devoid of standing water. However, permanent sampling stations were then haphazardly placed within these high salinity areas. Sampling stations depicted in Figure 1 are North Crystal Dig (NCD; N 36°44'18" W 98°16'18"), Clay Creek (CC; N 36°43'51" W 98°15'33") and South Crystal Dig (SCD; N 36°42'26" W 98°15'36").

### Experimental design

Temporal and spatial variation in algal biomass (i.e., Chlorophyll-*a *concentration) and physicochemical characteristics across the Great Salt Plains ecosystem were determined using a nested block design. Each of the three sampling stations consisted of a 20 × 10 m plot with three ground water wells (i.e., lysimeters) placed 10 m apart. Wells were constructed of 1.5-m lengths of PVC, permanently sealed at the bottom with perforations along the length of the pipe to allow for collection of ground water; each well was buried to the water table at a sediment depth of approximately 1.0 m and capped at the sediment surface to avoid rainwater inundation. Water samples were obtained from each of the wells for salinity determination and nutrient analyses (see below). Triplicate sediment samples for chlorophyll and moisture content were always taken at 0, 1 and 10 m distances in a west southwest direction from each well. All samples were collected monthly from July 2000 through July 2001.

### Sediment and ground water analyses

Traditional limnological and biochemical assays were used to determine Chlorophyll-*a *(Chl-*a*) and nutrient concentrations in ground water and sediment samples. Triplicate ground water samples were collected and analyzed for salt and nutrient content from each well at each station (NCD, CC and SCD; total n = 9 per station). To minimize degradation effects, samples for NH_4_^+ ^analysis were field-filtered (GF/F), placed on ice and immediately processed upon return to the laboratory as described by Parsons *et al*. [19]. Samples for NO_2_^- ^+ NO_3_^- ^and orthophosphate (PO_4_^3-^) were placed on ice, filtered and frozen upon return to the laboratory for later analysis. Concentrations of NO_2_^- ^+ NO_3_^- ^and PO_4_^3- ^were determined using standard colorimetric techniques, designed for the chemical analysis of seawater [19] and the ascorbic acid method [9], respectively. Estimates of algal abundance were made using Chl-*a *concentration as a proxy for relative biomass. Triplicate sediment cores (2-cm deep × 2.5-cm diameter) were obtained at distances of 0, 1 and 10 m from each well (total n = 27 per station) using a 60-mL disposable syringe with its end removed, transferred to 50-mL disposable centrifuge tubes and placed on ice. Samples were frozen upon return to the laboratory for later analysis. Sediment Chl extractions were performed in dim light by adding 8.0 mL N,N-dimethyl formamide (DMF) to each sediment tube. Samples were vortexed daily and allowed to extract for 7–10 d. At the end of the extraction period, samples were centrifuged at 10,000 rpm for 20 min and spectrophotometrically analyzed using the equations of Porra *et al*. [21]. Sediment samples were also assayed to determine and correct for phaeopigment content using traditional fluorometric methods after Lorenzen [16]. Sediment cores (8-cm deep × 1.3-cm diameter) were obtained using a 10-mL disposable syringe with its end removed, transferred to 15-mL pre-weighed disposable centrifuge tubes, placed on ice and frozen for later analysis. Moisture content of sediment-filled tubes was determined by slowly thawing, weighing (= wet weight) and drying sediment cores to a constant weight (= dry weight) for 7–10 d at 114°C. Percent moisture was then estimated by subtracting sediment dry weight from wet weight to determine the original water weight of each sample and expressing it as a percentage of wet weight.

### Algal species isolation and identification

Soil and ephemeral brine pool samples were collected from each of the three stations (i.e., NCD, CC and SCD; Fig. 1). Using the bottom of a sterile polystyrene petri dish (8.5-cm diam), 10 random cores from the top centimeter of salt plains soil were taken at each site and placed in a sterile plastic bag. Brine pool samples were collected in sterile Whirl-Pak^® ^bags. All soil and brine pool samples were placed on ice in the field and transferred to a cold room facility (~ 8°C) until initial isolations were performed the next day. Parallel sub-samples of soil (10 g) were suspended in 75 mL of sterile liquid medium or directly plated (~1 g soil) onto 1% agar plates (Bacto™) made with SP medium [10]. To maximize the diversity of algae isolated, we used three media with salinities of 10, 50 and 100 psu. Liquid and plate media with soil amendments were incubated under cool white light (60 μmol photons m^-2 ^s^-1^, 14:10 L/D) at a temperature range of 25–28°C. Once algal growth became visible (1–3 days), streak-plating was repeated to obtain unialgal cultures. Filamentous cyanobacteria were isolated using a phototactic purification method [25]. Using a Nikon E400 phase-contrast microscope, all algae were identified from live material to genus and when possible, species, under oil-immersion at 1000 × magnification. Chlorophyte algae were identified using Tomas [26] and Wehr and Sheath [29], while diatoms were identified using Cox [5], Round *et al*. [23] and Tomas [26]. Cyanobacteria were identified using the taxonomic keys of Komarek and Anagnostidis [15], Anagnostidis and Komarek [2] and Abed *et al*. [1].

### Soil temperature data

A Cox Tracer model CT1ED8 recording thermometer with an external sensor was used to measure soil surface temperature (T_soil_) at SCD from 19 June through 22 July and 31 July through 27 September 2001. Temperatures were automatically recorded at 15-min intervals. Air temperature (T_air_, °C) at 1.5-m height, solar radiation (W m^-2^) and wind speed (m s^-1^) at 10-m height were obtained from the Cherokee Oklahoma Mesonet Station located approximately 9.5-km northwest of SCD. The raw data were at 5-min intervals, so 15-min means were calculated for comparison to measured T_soil_. Because the latter were available for only part of the summer, Mesonet data were used to model T_soil _over a 4-month period (June – September). A direct multiple regression of the three Mesonet parameters explained 88.4% of the variance in measured T_soil_, and 90% of the residuals were within ± 5.2°C. The relationship was improved by using 1-h sliding means of solar irradiance with a 3-h lag, which resulted in a subjectively acceptable model: predicted T_soil _= 0.924T_air _+ 0.010lagsolar - 0.383wind + 5.743 (n = 8699, r^2 ^= 0.937, 90% of residuals within ± 3.5°C). T_air_, lagged solar and wind speed explained 85.4%, 7.5% and 0.8% of the variance in T_soil_, respectively.

### Statistics

Analyses were performed on biotic and abiotic data collected from the three permanent sampling stations (i.e., NCD, CC and SCD). All statistical tests were considered significant at the level of P < 0.05. Sampling dates with missing data were excluded to prevent bias. A one-way ANOVA was performed to test for mean differences among wells for each site. A Tukey pair-wise comparison for each well was conducted for each site between chlorophyll and wells to determine the relative order of variables.

Since samples from each well were potentially intercorrelated, a Principal Components Analysis (PCA) was conducted to summarize the variables into smaller, uncorrelated subsets. Stepwise regressions were conducted on 1) chlorophyll (response variable) versus each predictor variable for each site and 2) chlorophyll versus predictor variables and principal components. Data were log-transformed and a Discriminant Function Analysis was conducted to identify those sites that were most alike and to identify variables most useful for distinguishing among groups.

## Competing interests

The author(s) declare that they have no competing interests.

## Authors' contributions

KMM participated in the design and implementation of this study, collected field data and drafted the largest portion of this manuscript. AEK was responsible for isolation and identification of algal strains and drafted a portion of this manuscript. CSM and JWM performed statistical analyses and drafted a portion of this manuscript. WJH conceived of this study, participated in its design and coordination, contributed soil temperature data and drafted a portion of this manuscript. All authors read and approved this final manuscript.
